# Associations of modifiable dementia risk factors with dementia and cognitive decline: evidence from three prospective cohorts

**DOI:** 10.3389/fpubh.2025.1529969

**Published:** 2025-01-15

**Authors:** Mengzhao Wang, Changming Fan, Yanbai Han, Yifei Wang, Hejia Cai, Wanying Zhong, Xin Yang, Zhenshan Wang, Hongli Wang, Yiming Han

**Affiliations:** ^1^College of Physical Education and Health, Guangxi Normal University, Guilin, China; ^2^Department of Physical Education, Hebei University of Environmental Engineering, Qinhuangdao, China; ^3^Outdoor Sports Academy, Guilin Tourism University, Guilin, China

**Keywords:** modifiable risk factors, coexistence, dementia, cognitive decline, cohort study

## Abstract

**Objective:**

This study aims to assess the relationship between modifiable dementia risk factors and both dementia and cognitive decline.

**Methods:**

Data were obtained from the Health and Retirement Study (HRS) [2008–2020], the China Health and Retirement Longitudinal Study (CHARLS) [2011–2020], and the English Longitudinal Study of Ageing (ELSA) [2010–2020]. After adjusting for confounding factors, multivariable logistic regression was utilized to analyze the relationship between modifiable dementia risk factors and dementia, while multivariable linear regression was employed to examine the relationship between these risk factors and cognitive decline. Additionally, the Cox proportional hazards model was used to assess the relationship between the number of risk factor events, clusters, and dementia risk.

**Results:**

A total of 30,113 participants from HRS, CHARLS, and ELSA were included (44.6% male, mean age 66.04 years), with an average follow-up period of 7.29 years. A low education level was significantly associated with an increased risk of dementia and accelerated cognitive decline (Overall, OR = 2.93, 95% CI: 2.70–3.18; Overall, *β* = −0.25, 95% CI: −0.60 to-0.55). The presence of multiple dementia risk factors correlated with a higher dementia risk; Specifically, compared with more than 5 risk factor events, both having no dementia risk factors and having only one dementia risk factor were associated with a significantly lower risk of dementia (Overall, HR = 0.15, 95% CI: 0.11–0.22, HR = 0.22, 95% CI: 0.18–0.25). Compared to the group with no coexistence of risk factors, the clusters of excessive alcohol, diabetes, vision loss, and hearing loss (HR = 4.11; 95% CI = 3.42–4.95; *p* < 0.001); excessive alcohol, vision loss, smoking, and hearing loss (HR = 5.18; 95% CI = 4.30–6.23; *p* < 0.001); and excessive alcohol, obesity, diabetes, and smoking (HR = 5.96; 95% CI = 5.11–6.95; p < 0.001) were most strongly associated with dementia risk.

**Conclusion:**

Among the 11 risk factors, educational attainment has the greatest impact on dementia risk and cognitive decline. A dose–response relationship exists between the number of modifiable risk factor events and dementia risk. The coexistence of multiple risk factors is associated with dementia risk, and these associations vary by risk factor cluster.

## 1 Introduction

Dementia represents a syndrome marked by swift cognitive deterioration, along with a decline in the capacity for daily living. From a clinical perspective, dementia can be categorized into early and late stages; during the initial phase, individuals might display only subtle cognitive decline, exerting negligible effects on daily activities (1). As the condition advances, individuals may undergo pronounced cognitive impairments and a loss of daily functioning, which greatly impacts both the patients and their caregivers, imposing a significant socioeconomic burden (2). With the rise in life expectancy and an expanding older adult demographic, the number of individuals affected by dementia has exceeded 57 million, significantly impacting social and economic frameworks, thereby establishing it as a prominent global health challenge (3). The principal manifestations of dementia include cognitive decline and functional impairment. The efficacy of pharmacological interventions for dementia remains restricted, necessitating considerable medical and caregiving resources. Therefore, the prevention and postponement of dementia’s onset have emerged as crucial focal points in contemporary research (4).

The Lancet Dementia Commission has recognized 14 modifiable risk factors for dementia through systematic reviews and meta-analyses of high-caliber studies, potentially linked to more than 45% of dementia cases (5). This discovery opens up promising avenues for interventions designed to prevent or delay dementia via targeted strategies (6). Certain modifiable risk factors may not exhibit a strong correlation with dementia yet show a significant relationship with cognitive decline, underscoring their potential role in dementia prevention (7, 8). A prospective cohort study utilizing the UK Biobank suggested that enhancing educational attainment during middle to late life could markedly diminish the risk of developing dementia in later years (9). Research conducted in South Korea revealed that women experiencing depression had a substantially elevated risk of dementia compared to their non-depressed counterparts (10).

Most prior research has primarily focused on the relationship between single modifiable risk factors and either dementia or cognitive abilities (11, 12). However, risk factors often coexist and mutually influence one another across different life stages, such as depression and social isolation. Social isolation may elevate the risk of depression, while early depression can exacerbate the likelihood of social isolation, illustrating a bidirectional relationship between these two factors (13–15). Limited research has explored the interplay between multiple coexisting modifiable risk factors and their effects on dementia and cognitive abilities across the lifespan. Consequently, this study aims to explore the association between modifiable dementia risk factors and both dementia and cognitive decline in individuals over the age of 50, while also examining the relationship between the number of these risk factor events, the coexistence of multiple risk factor events, and dementia risk.

## 2 Methods

### 2.1 Study design and population

HRS, CHARLS, and ELSA represent national prospective cohort studies that encompass older adults in the United States, China, and England, respectively. To guarantee comparability in dementia and cognitive function across the three cohorts while maintaining a consistent time frame, this study employed the 9th wave of the HRS survey (2008), the 1st wave of the CHARLS survey (2011), and the 4th wave of the ELSA survey (2010) as baseline data. The 15th wave of the HRS survey (2020), the 5th wave of the CHARLS survey (2020), and the 9th wave of the ELSA survey (2020) functioned as the final follow-up assessments. Ethical approval for HRS, CHARLS, and ELSA was secured from the institutional review boards of the University of Michigan, Peking University, and the London Multicentre Research Ethics Committee, respectively. All participants provided their written informed consent.

This study identified 11 out of the 14 modifiable risk factors (low education level, hearing loss, hypertension, smoking, obesity, depression, physical activity, diabetes, alcohol consumption, social isolation, and vision loss) as independent variables by analyzing the matching levels of modifiable dementia risk factors across the three cohorts. The reasons for excluding the three risk factors from this study include: data on traumatic brain injury (TBI) was unavailable in ELSA; air pollution data could not be acquired for all three countries; and high LDL cholesterol data exhibited excessive missing values from the baseline sampling in CHARLS.

**Figure 1:**
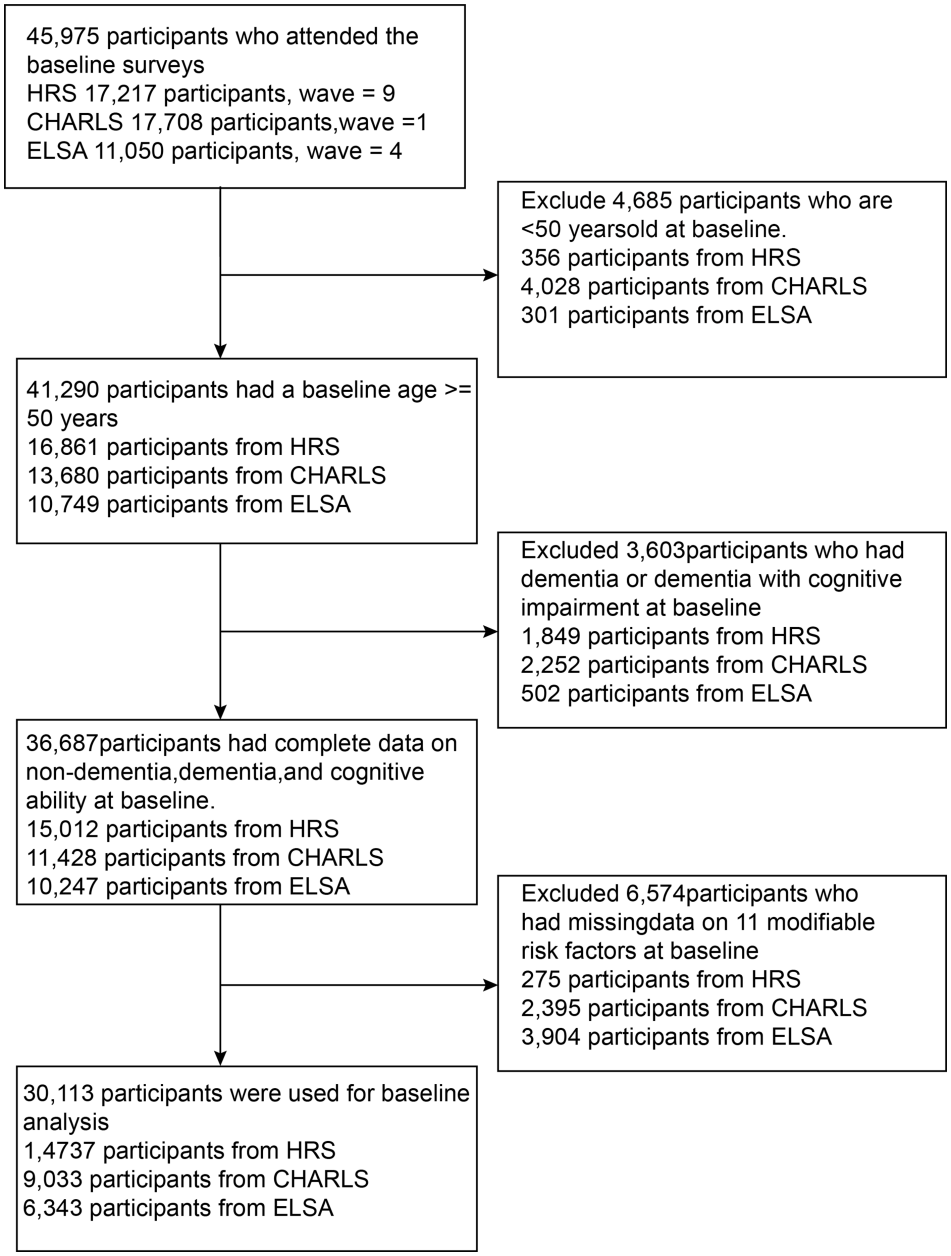
Flow chart of study participant selection.

Figure 1 shows the selection process of the study population. The HRS sample included 17,217 participants recruited at baseline, with 2,480 excluded (356 under 50 years, 1,849 with dementia or unable to provide cognitive and dementia status, and 275 without baseline modifiable dementia risk factors). The CHARLS sample included 17,708 participants recruited at baseline, with 8,675 excluded (4,028 under 50 years, 2,252 with dementia, and 2,395 without baseline modifiable dementia risk factors). The ELSA sample consisted of 11,050 participants, with 4,646 excluded (301 under 50 years, 502 with dementia, and 3,904 without baseline modifiable dementia risk factors). Ultimately, a total of 30,113 participants (14,737 from HRS, 9,033 from CHARLS, and 6,343 from ELSA) were included in the study.

### 2.2 Modifiable dementia risk factors

The risk factors delineated in this study are as follows: Education level is categorized according to the International Standard Classification of Education (ISCE 2011), with individuals completing junior high school or below classified as having a low educational attainment (16, 17). Hearing loss is characterized by either the use of hearing aids or self-reported poor auditory ability (18, 19). In alignment with prior studies, hypertension is defined as having a systolic/diastolic blood pressure of ≥140/90 mmHg or self-reported hypertension (20). Smoking is determined by self-reported current smoking status. Obesity is defined as a body mass index (BMI) of ≥30 kg/m^2^.

Depression is evaluated using various scales: the CESD-10 questionnaire for CHARLS and the CESD-8 questionnaire for HRS and ELSA, with depression operationally defined as a CESD-10 score of ≥10 in CHARLS and a CESD-8 score of ≥3 in HRS and ELSA (21, 22). Physical inactivity is defined as engaging in vigorous or moderate activities for fewer than three days per week, with each session lasting at least 10 min (23). Diabetes is characterized by blood tests indicating a fasting blood glucose level of ≥126 mg/dL or HbA1c of ≥6.5%, or by self-reported physician-diagnosed diabetes (24). Excessive alcohol consumption is defined as self-reported drinking frequency exceeding half the week, encompassing consumption 4–6 days per week, daily, twice a day, or more than twice daily (25).

To evaluate social isolation, participants were assigned a social isolation score based on the following criteria: (i) unmarried, (ii) living alone, (iii) contact with children less than once a week, (iv) contact with parents, relatives, or friends less than once a week, and (v) no participation in any groups, clubs, or organizations in the past month (HRS, CHARLS) or past year (ELSA). The scoring ranges from 0 to 5, with higher scores indicating increased levels of social isolation. Based on prior studies, a score of ≥2 is designated as indicating social isolation (26, 27). To evaluate vision loss, in CHARLS, it is defined as a self-reported poor score in nearsightedness or farsightedness, whereas in HRS and ELSA, it is characterized as self-reported vision described as “blind” or “poor” (28–30). We defined the number of modifiable dementia risk factor events as a continuous variable ranging from 0 to 11 and as a categorical variable with the following categories: 0, 1, 2, 3, 4, 5, and > 5. The definition of multiple risk factor coexistence is the presence of at least 2 out of 11 conditions. Participants with 0 or 1 condition are defined as having no coexistence and serve as the reference group in the analysis (31).

### 2.3 Cognitive decline and dementia

In all three cohorts, cognitive function is predominantly assessed using tests that evaluate executive function, orientation, and memory. Elevated test scores signify enhanced cognitive abilities. The rate of cognitive decline is assessed by calculating z-scores. The z-score for each cognitive domain is computed by subtracting the baseline mean score and dividing by the baseline standard deviation (SD). The overall cognitive z-score is obtained by averaging the z-scores of the three cognitive test domains, followed by standardization through subtracting the baseline mean and dividing by the baseline SD. A global cognitive z-score of-1 indicates that the score is 1 SD lower than the mean cognitive score at baseline (32, 33). This study focuses exclusively on the rate of overall cognitive decline among participants.

In HRS, cognitive function scores were utilized to evaluate dementia, encompassing both memory and executive function domains, with a score range from 0 to 27. A score of ≤6 is deemed indicative of dementia (34). In ELSA and CHARLS, dementia is defined as the coexistence of cognitive impairment (involving two or more impaired cognitive function domains) and functional impairment (35–38). Cognitive impairment is characterized by scores in two or more cognitive function domains that are equal to or fall below 1.5 standard deviations beneath the mean, standardized for a population aged 50–80 with comparable education levels, akin to the criteria for cognitive impairment without dementia (CIND) (35). Functional impairment is described as challenges in performing one or more activities of daily living, which include moving around the room, dressing, bathing, eating, getting in and out of bed, and using the toilet (37).

### 2.4 Covariates

The covariates considered in this study encompass age, gender, history of stroke, and heart disease. Age is determined by subtracting the birth year from the survey year, whereas stroke and heart disease are self-reported by the participants.

### 2.5 Statistical analysis

For descriptive statistics, continuous variables are presented as mean (SD), while categorical variables are represented as counts (percentages). Multiple imputation was utilized to address missing data for physical activity variables in the CHARLS cohort. Multivariable logistic regression was employed to assess the association between dementia (dependent variable) and modifiable dementia risk factors (independent variables). The Cox proportional hazards model was used to assess the relationship between the coexistence of modifiable dementia risk factors and dementia risk. Additionally, multivariable linear regression was applied to determine the relationship between modifiable risk factors and cognitive decline. We utilized Population Attributable Fractions (PAF) to quantify the contribution of controlling individual risk factors to reducing the burden of dementia.

**Figure 2:**
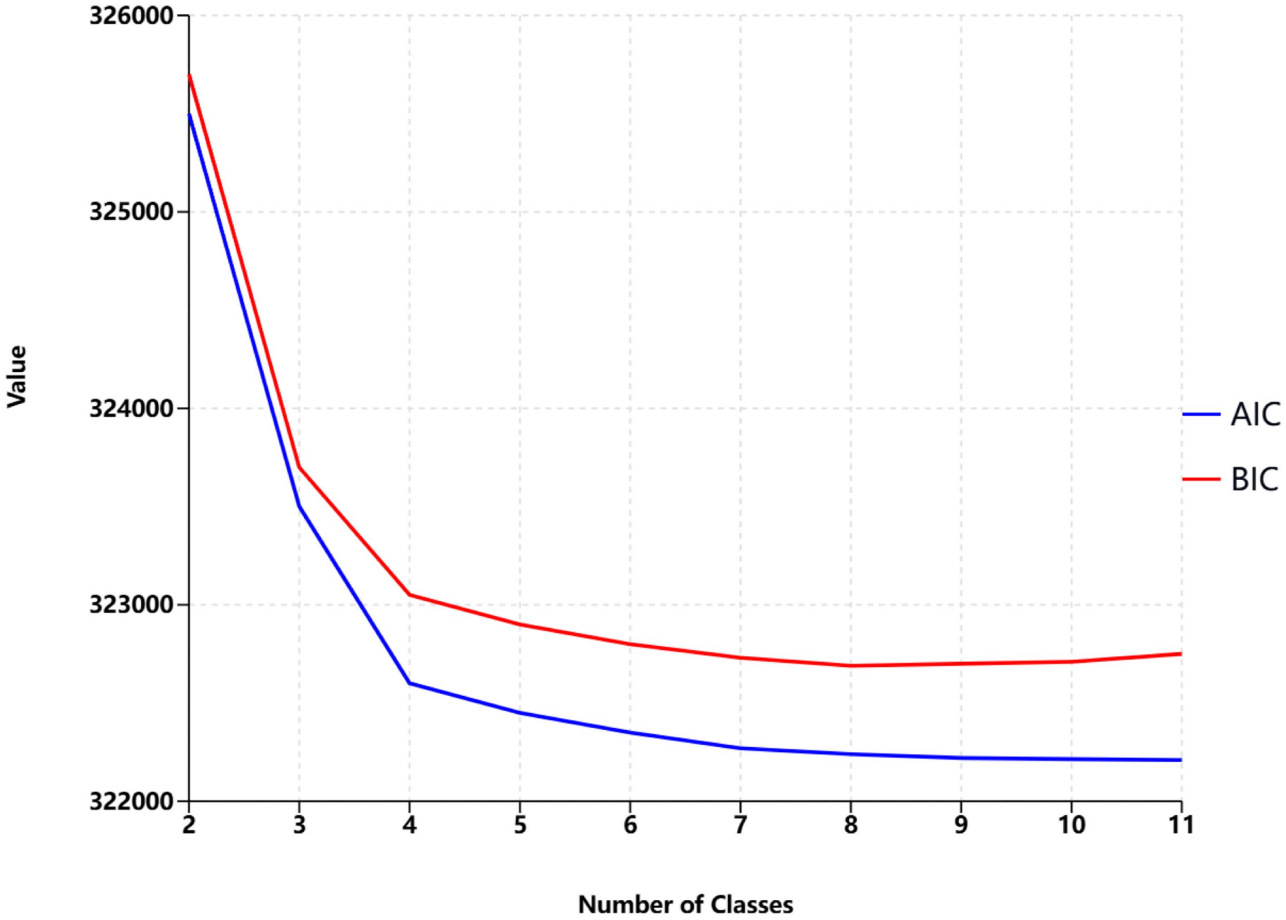
Optimal number of classes based on AIC and BIC values.

**Figure 3:**
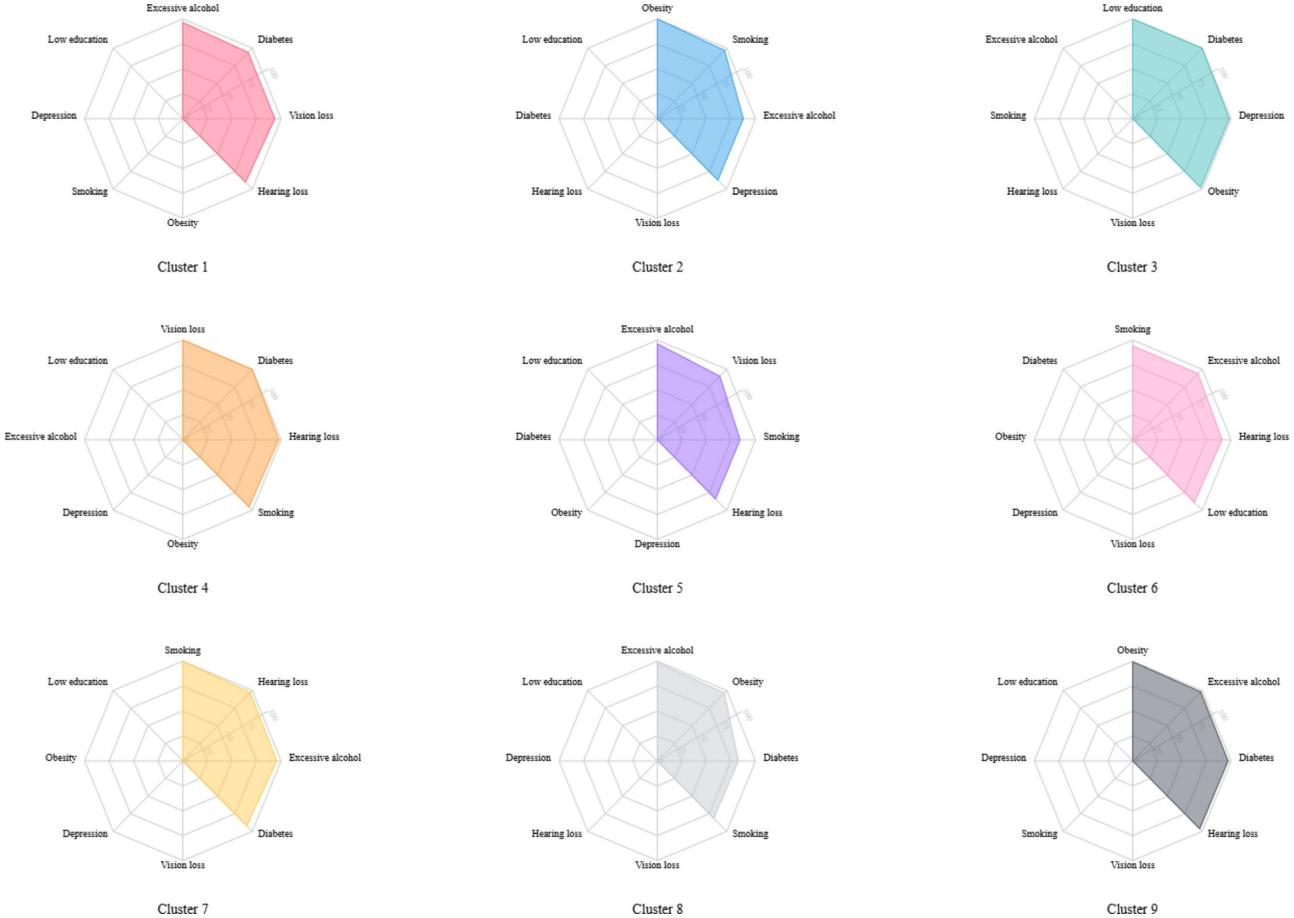
The proportion of risk factors in different clusters.

Latent class analysis was used to identify clusters of risk factors, assigning each participant with multiple concurrent risk factors to a non-overlapping cluster while allowing health conditions to contribute to multiple clusters with different probabilities. A random training sample of participants with multiple risk factors was used to determine the optimal number of clusters, followed by an estimation of the association between dementia risk factor clusters and dementia risk. Statistical metrics were generated for multiple latent class analysis models of clustering solutions, with the optimal number of clusters determined by the Bayesian Information Criterion adjusted for sample size and by restricting the minimum cluster size to more than 5% of the sample. The optimal number of clusters was determined to be 4 (see Figure 2), A total of nine clusters were included in this study (see Figure 3). To validate the reasonableness of selecting the 4-cluster model as the best-fitting solution, we calculated the posterior probability distribution based on BIC values using the formula: The posterior probabilities for k = 1, k = 2, k = 3, k = 4, k = 5, k = 6 were calculated as 0, 0.016, 0.313, 0.329, and 0.342, respectively. The results show that the 4-cluster model exhibits a relatively high posterior probability (0.313). While the posterior probabilities for the 5-and 6-cluster models were slightly higher, the differences were not significant. Considering model simplicity and interpretability, the selection of the 4-cluster model as the optimal solution is reasonable. Each cluster was characterized by the four most influential risk factors specific to that cluster (see Table 1). In addition, we conducted PAF analyses for each cluster to quantify the potential benefits of interventions.

**Table 1:** Characteristics of latent class analysis for the overall participants.

Cluster	% of sample (n = 30,113)	Risk factor 1	Risk factor 2	Risk factor 3	Risk factor 4
1	6.1	Excessive alcohol (96.5%)	Diabetes (94.0%)	Vision loss (93.6%)	Hearing loss (90.3%)
2	5.7	Obesity (100.0%)	Smoking (96.9%)	Excessive alcohol (87.6%)	Depression (87.3%)
3	6.2	Low education (100.0%)	Diabetes (100.0%)	Depression (98.2%)	Obesity (97.8%)
4	12.6	Vision loss (100.0%)	Diabetes (99.9%)	Hearing loss (97.6%)	Smoking (95.2%)
5	5.2	Excessive alcohol (96.3%)	Vision loss (90.2%)	Smoking (84.3%)	Hearing loss (83.9%)
6	11	Smoking (94.2%)	Excessive alcohol (93.9%)	Hearing loss (91.0%)	Low education (88.7%)
7	7.2	Smoking (100.0%)	Hearing loss (97.0%)	Excessive alcohol (95.6%)	Diabetes (92.0%)
8	7.1	Excessive alcohol (98.6%)	Obesity (96.5%)	Diabetes (82.5%)	Smoking (81.4%)
9	13.1	Obesity (99.4%)	Excessive alcohol (97.9%)	Diabetes (97.0%)	Hearing loss (96.4%)

All statistical analyses were two-sided, with *p* < 0.05 considered statistically significant. Analyses were conducted using Stata version 17 (Stata Corp, College Station, Texas) and R version 4.3.2 (R Foundation for Statistical Computing, Vienna, Austria).

## 3 Results

### 3.1 Baseline characteristics of the study participants

Table 2 presents the baseline characteristics of participants from HRS, CHARLS, ELSA, and the overall cohort. Overall, participants included in the study had a mean age of 66.04 years, with 44.6% being male. The prevalence of low education (42.5%), physical inactivity (40.1%), hypertension (57.0%), and social isolation (37.1%) was notably high. In HRS, there were 14,737 participants (mean age 68.89 years, 40.8% male); in CHARLS, there were 9,033 participants (mean age 62.01 years, 49.0% male); and in ELSA, there were 6,343 participants (mean age 65.13 years, 47.1% male). Among the three prospective cohorts, HRS participants had the highest prevalence of hypertension (61.9%), while CHARLS participants reported the highest rates of low education (90.2%), depression (36.9%), and vision loss (37.0%). The obesity rate was lowest in CHARLS (4.7%), whereas ELSA participants exhibited high rates of physical inactivity (68.9%), excessive alcohol consumption (32.4%), and social isolation (57.3%). Overall, among 27,560 follow-up participants, 3,400 cases of dementia were reported over an average follow-up period of 7.29 years. During an average follow-up of 6.84 years in HRS, 1,293 cases of dementia occurred among 13,213 participants. In CHARLS, during an average follow-up of 7.38 years, 1,438 cases of dementia were reported among 8,427 participants. In ELSA, during an average follow-up of 8.17 years, 669 cases of dementia occurred among 5,920 participants.

**Table 2:** Participants’ baseline characteristics.

	Overall	HRS	CHARLS	ELSA
Sample size, *n*	30,113	14,737	9,033	6,343
Age [mean (SD)]	66.04 (9.66)	68.89 (9.83)	62.01 (8.13)	65.13 (9.04)
Male (%)	13,428 (44.6)	6,012 (40.8)	4,428 (49.0)	2,988 (47.1)
stroke (%)	1,555 (5.2)	1,144 (7.8)	226 (2.5)	185 (2.9)
Heart disease (%)	5,653 (18.8)	3,557 (24.1)	1,168 (12.9)	928 (14.6)
Less education (%)	12,784 (42.5)	2,709 (18.4)	8,147 (90.2)	1928 (30.4)
Vision loss (%)	6,528 (21.7)	3,019 (20.5)	3,345 (37.0)	164 (2.6)
Hypertension (%)	17,151 (57.0)	9,119 (61.9)	4,648 (51.5)	3,384 (53.4)
Smoking (%)	5,504 (18.3)	1910 (13.0)	2,828 (31.3)	766 (12.1)
Obesity (%)	6,945 (23.1)	4,593 (31.2)	426 (4.7)	1926 (30.4)
Depression (%)	7,420 (24.6)	2,980 (20.2)	3,329 (36.9)	1,111 (17.5)
Physical inactivity (%)	12,071 (40.1)	4,213 (28.6)	3,488 (38.6)	4,370 (68.9)
Excessive alcohol (%)	5,199 (17.3)	1907 (12.9)	1,239 (13.7)	2053 (32.4)
Diabetes (%)	4,763 (15.8)	2,998 (20.3)	1,240 (13.7)	525 (8.3)
Social isolation (%)	11,184 (37.1)	4,132 (28.0)	3,415 (37.8)	3,637 (57.3)
Hearing loss (%)	3,515 (11.7)	1918 (13.0)	1,356 (15.0)	241 (3.8)

### 3.2 Association between modifiable dementia risk factors and dementia

Multivariable logistic regression analysis was conducted to compare the direction and magnitude of associations between modifiable dementia risk factors and dementia among the overall cohort, as well as participants from HRS, CHARLS, and ELSA. Low education level, depression, and hearing loss were significantly associated with an increased risk of dementia. In the overall cohort, hypertension was found to promote the risk of dementia, although the statistical significance was weak (OR = 1.07; 95% CI = 0.99–1.16; *p* = 0.084), with similar results in HRS and ELSA. In contrast, CHARLS exhibited a stronger association (OR = 1.14; 95% CI = 1.00–1.28; *p* = 0.043). Smoking also increased the risk of dementia in the overall cohort, but the statistical significance was weak (OR = 1.06; 95% CI = 0.96–1.17; *p* = 0.252). However, stronger associations were observed in HRS, CHARLS, and ELSA (OR = 1.28; 95% CI = 1.05–1.54; *p* = 0.013; OR = 1.25; 95% CI = 1.05–1.47; p = 0.01; OR = 1.81; 95% CI = 1.44–2.26; *p* < 0.001). Notably, a lack of physical activity was linked to an increased risk of dementia across the overall cohort, although the statistical significance was weak (OR = 1.06; 95% CI = 0.98–1.15; *p* = 0.136). The results for HRS and CHARLS were consistent; however, a stronger association was observed in ELSA (OR = 1.83; 95% CI = 1.47–2.29; p < 0.001). Vision loss was also associated with an increased risk of dementia in the overall cohort, although the statistical significance was weak (OR = 1.07; 95% CI = 0.98–1.17; *p* = 0.141). The results were consistent with HRS; however, CHARLS and ELSA showed stronger associations (OR = 1.26; 95% CI = 1.11–1.43; p < 0.001; OR = 1.59; 95% CI = 1.04–2.38; *p* = 0.028). Additionally, obesity and diabetes were significantly associated with an increased risk of dementia in the overall cohort (see Table 3). In addition, we performed PAF analyses on the aggregated cohort from three countries to assess the potential benefits of controlling various risk factors. Among these, low educational attainment (PAF = 45.1%), depression (PAF = 17.8%), social isolation (PAF = 6.3%), and hearing loss (PAF = 5.9%) were identified as the four risk factors with the highest potential contributions to the burden of dementia. Association between modifiable dementia risk factors and cognitive decline.

**Table 3:** Association between modifiable risk factors and dementia: Multinomial logistic regression analysis.

	Overall		HRS		CHARLS		ELSA	
	OR (95% CI)	*p*	OR (95% CI)	*p*	OR (95% CI)	*p*	OR (95% CI)	*p*
Age	1.03 (1.02, 1.03)	<0.001	1.07 (1.07, 1.08)	<0.001	0.98 (0.98, 0.99)	<0.001	1.02 (1.01, 1.03)	0.003
Sex	0.73 (0.67, 0.79)	<0.001	0.92 (0.80, 1.05)	0.2	0.46 (0.39, 0.54)	<0.001	1.16 (0.98, 1.38)	0.093
Low education	**2.93 (2.70, 3.18)**	**<0.001**	4.05 (3.56, 4.61)	<0.001	4.59 (3.19, 6.87)	<0.001	1.42 (1.18, 1.70)	<0.001
Vision loss	1.07 (0.98, 1.17)	0.141	0.88 (0.74, 1.04)	0.13	1.26 (1.11, 1.43)	<0.001	1.59 (1.04, 2.38)	0.028
Hypertension	1.07 (0.99, 1.16)	0.084	1.05 (0.91, 1.20)	0.523	1.14 (1.00, 1.28)	0.043	1.13 (0.95, 1.36)	0.175
Smoking	1.06 (0.96, 1.17)	0.252	1.28 (1.05, 1.54)	0.013	1.25 (1.05, 1.47)	0.01	1.81 (1.44, 2.26)	<0.001
Obesity	**1.11 (1.01, 1.22)**	**0.028**	0.91 (0.79, 1.05)	0.182	1.34 (1.03, 1.72)	0.025	1.81 (1.51, 2.15)	<0.001
Depression	**1.88 (1.73, 2.03)**	**<0.001**	1.54 (1.33, 1.78)	<0.001	2.06 (1.82, 2.32)	<0.001	2.27 (1.87, 2.74)	<0.001
Physical inactivity	1.06 (0.98, 1.15)	0.136	1.08 (0.94, 1.23)	0.282	0.89 (0.78, 1.01)	0.064	1.83 (1.47, 2.29)	<0.001
Excessive alcohol	0.79 (0.71, 0.89)	<0.001	0.74 (0.59, 0.92)	0.008	0.80 (0.64, 0.99)	0.045	0.81 (0.67, 0.99)	0.04
Diabetes	**1.18 (1.07, 1.30)**	**0.001**	1.37 (1.18, 1.59)	<0.001	1.12 (0.94, 1.32)	0.204	1.08 (0.82, 1.40)	0.597
Social isolation	**1.18 (1.09, 1.27)**	**<0.001**	1.26 (1.10, 1.43)	0.001	1.17 (1.04, 1.33)	0.011	0.93 (0.78, 1.10)	0.393
Hearing loss	**1.54 (1.39, 1.71)**	**<0.001**	1.30 (1.10, 1.53)	0.002	1.52 (1.29, 1.77)	<0.001	1.64 (1.14, 2.31)	0.006

The references for each variable were female, normal education, normal vision, no hypertension, no smoking, no obesity, no depression, normal physical activity, non-excessive alcohol consumption, no diabetes, no social isolation, and normal hearing. Multivariable models were adjusted for age, sex, stroke and heart disease. Bold values indicate a significant positive association between risk factors and incident dementia in the overall population.

In the overall cohort, as well as in HRS, CHARLS, and ELSA, low education level, smoking, depression, diabetes, vision loss, and hearing loss were associated with accelerated cognitive decline. In HRS and ELSA, a lack of physical activity was related to accelerated cognitive decline, whereas in the overall cohort and CHARLS, a lack of physical activity was associated with a slowdown in cognitive decline. In the overall cohort, obesity was found to promote a slowdown in cognitive decline, although the statistical significance was weak (*β* = 0.01; 95% CI = 0.00–0.05; *p* = 0.1). Hypertension was significantly associated with accelerated cognitive decline, and excessive alcohol consumption was linked to a slowdown in cognitive decline. Additionally, social isolation was also associated with accelerated cognitive decline (see Table 4).

**Table 4:** Relationship between modifiable risk factors and cognitive decline: Linear regression with multiple variables analysis.

	Overall		HRS		CHARLS		ELSA	
	*β* (95% CI)	*p*	*β* (95% CI)	*p*	*β* (95% CI)	*p*	*β* (95% CI)	*p*
Age	−0.30 (−0.04, −0.04)	<0.001	−0.30 (−0.04, −0.03)	<0.001	−0.20 (−0.03, −0.03)	<0.001	−0.34 (−0.03, −0.03)	<0.001
Sex	0.05 (0.10, 0.15)	<0.001	−0.01 (−0.05, 0.02)	0.326	0.23 (0.45, 0.56)	<0.001	−0.11 (−0.23, −0.15)	<0.001
Low education	**−0.25 (−0.60, −0.55)**	**<0.001**	−0.25 (−0.75, −0.66)	<0.001	−0.21 (−0.86, −0.72)	<0.001	−0.12 (−0.27, −0.17)	<0.001
Vision loss	**−0.05 (−0.17, −0.11)**	**<0.001**	0.04 (0.06, 0.14)	<0.001	−0.04 (−0.14, −0.05)	<0.001	−0.05 (−0.40, −0.14)	<0.001
Hypertension	**−0.02 (−0.07, −0.02)**	**0.001**	−0.02 (−0.07, 0.00)	0.059	0.00 (−0.05, 0.04)	0.827	0.02 (−0.02, 0.07)	0.215
Smoking	**−0.04 (−0.15, −0.08)**	**<0.001**	−0.05 (−0.21, −0.11)	<0.001	−0.05 (−0.18, −0.07)	<0.001	−0.04 (−0.16, −0.04)	0.001
Obesity	0.01 (0.00, 0.05)	0.1	0.00 (−0.04, 0.03)	0.742	0.03 (0.06, 0.26)	0.001	0.00 (−0.05, 0.04)	0.8
Depression	**−0.10 (−0.30, −0.24)**	**<0.001**	−0.08 (−0.27, −0.18)	<0.001	−0.09 (−0.24, −0.15)	<0.001	−0.07 (−0.21, −0.10)	<0.001
Physical inactivity	0.10 (0.20, 0.25)	<0.001	−0.03 (−0.12, −0.04)	<0.001	0.04 (0.04, 0.13)	<0.001	−0.04 (−0.12, −0.03)	<0.001
Excessive alcohol	0.09 (0.23, 0.29)	<0.001	0.04 (0.09, 0.19)	<0.001	0.00 (−0.07, 0.06)	0.945	0.04 (0.03, 0.11)	0.001
Diabetes	**−0.06 (−0.22, −0.15)**	**<0.001**	−0.05 (−0.19, −0.10)	<0.001	−0.02 (−0.13, −0.01)	0.023	−0.03 (−0.17, −0.03)	0.006
Social isolation	**−0.03 (−0.10, −0.05)**	**<0.001**	−0.02 (−0.09, −0.02)	0.006	−0.09 (−0.24, −0.15)	<0.001	0.01 (−0.02, 0.06)	0.235
Hearing loss	**−0.07 (−0.30, −0.22)**	**<0.001**	−0.04 (−0.20, −0.09)	<0.001	−0.05 (−0.23, −0.11)	<0.001	−0.05 (−0.33, −0.12)	<0.001

Multivariable models were adjusted for age, sex, stroke and heart disease. *R*^2^ values for each model: Overall = 0.22, HRS = 0.21, CHARLS = 0.23, ELSA = 0.20. Bold values indicate a significant negative association between risk factors and cognitive function in the overall population.

### 3.3 Correlation between the number of modifiable dementia risk factors and dementia risk

Multiple potentially modifiable risk factors for dementia frequently coexist in the older adult population. On average, participants in the entire cohort had 3.35 risk factors, while HRS participants had 2.61 risk factors, CHARLS participants had 4.67 risk factors, and ELSA participants had 3.14 risk factors. When the number of modifiable risk factors for dementia was considered a continuous variable, for each additional risk factor in the entire cohort, the risk of dementia increased by 39% (HR = 1.32; 95% CI = 1.30–1.35; *p* < 0.001). In the HRS, each additional risk factor was associated with a 39% increased risk of dementia (HR = 1.39; 95% CI = 1.34–1.43; *p* < 0.001). In CALLS, each additional risk factor was associated with a 25% increased risk of dementia (HR = 1.25; 95% CI = 1.21–1.29; *p* < 0.001). In ELSA, each additional risk factor corresponded to a 51% increased risk of dementia (HR = 1.51; 95% CI = 1.44–1.59; *p* < 0.001). When the number of modifiable risk factors for dementia was considered a categorical variable, there was a dose–response relationship with fewer risk factors being associated with a lower risk of dementia compared with more than five risk factors, both overall and in the three cohorts separately (see Table 5).

**Table 5:** Relationship between number of modifiable risk factors and dementia: Cox multivariate analysis.

	Overall		HRS		CHARLS		ELSA	
Number of modifiable dementia risk factors (0–11)	Mean (SD)		Mean (SD)		Mean (SD)		Mean (SD)	
	3.35 (1.77)		2.61 (1.52)		4.67 (1.55)		3.14 (1.53)	

Number of modifiable dementia risk factors (continuous)	HR (95% CI)	*p*	HR (95% CI)	*p*	HR (95% CI)	*p*	HR (95% CI)	*p*
	1.32 (1.30, 1.35)	<0.001	1.39 (1.34, 1.43)	<0.001	1.25 (1.21, 1.29)	<0.001	1.51 (1.44, 1.59)	<0.001

Number of modifiable dementia risk factors (categorical)	HR (95% CI)	*p*	HR (95% CI)	*p*	HR (95% CI)	*p*	HR (95% CI)	*p*
**5+**	1.00 (Reference)		1.00 (Reference)		1.00 (Reference)		1.00 (Reference)	
**5**	**0.73 (0.66, 0.81)**	**<0.001**	**0.68 (0.54, 0.86)**	**<0.001**	**0.76 (0.67, 0.87)**	**<0.001**	**0.69 (0.54, 0.88)**	**<0.001**
**4**	0.55 (0.50, 0.61)	<0.001	0.52 (0.42, 0.64)	<0.001	0.57 (0.50, 0.66)	<0.001	0.42 (0.33, 0.54)	<0.001
**3**	0.39 (0.35, 0.43)	<0.001	0.36 (0.29, 0.45)	<0.001	0.42 (0.35, 0.50)	<0.001	0.27 (0.21, 0.35)	<0.001
**2**	0.26 (0.23, 0.29)	<0.001	0.23 (0.19, 0.29)	<0.001	0.34 (025, 0.45)	<0.001	0.15 (0.11, 0.20)	<0.001
**1**	0.22 (0.18, 0.25)	<0.001	0.19 (0.15, 0.24)	<0.001	0.05 (0.01, 0.38)	<0.001	0.15 (0.11, 0.22)	<0.001
**0**	0.15 (0.11, 0.22)	<0.001	0.13 (0.09, 0.19)	<0.001			0.08 (0.03, 0.20)	0.003

Adjusted for age, sex, stroke and heart disease. There are no participants without risk factors in CHARLS. Bold values indicate that a greater number of coexisting risk factors is associated with higher dementia risk.

### 3.4 Association of clusters of modifiable dementia risk factors with dementia risk

In all clusters, the four risk factors represent the optimal number of clusters, with the cluster formed by low education, diabetes, depression, and obesity showing no association with dementia risk (HR = 0.97; 95% CI = 0.76–1.24; *p* = 0.81). All other clusters were associated with an increased risk of dementia. Compared to the group with no coexistence of risk factors, the clusters of excessive alcohol, diabetes, vision loss, and hearing loss (HR = 4.11; 95% CI = 3.42–4.95; *p* < 0.001); excessive alcohol, vision loss, smoking, and hearing loss (HR = 5.18; 95% CI = 4.30–6.23; *p* < 0.001); and excessive alcohol, obesity, diabetes, and smoking (HR = 5.96; 95% CI = 5.11–6.95; *p* < 0.001) were most strongly associated with dementia risk (see Table 6). In addition, we conducted PAF analyses for each cluster within the overall cohort to assess the potential benefits of controlling risk factors in each cluster. The clusters with the highest potential contributions to the dementia disease burden were excessive alcohol, obesity, diabetes, and smoking (PAF = 58.6%), excessive alcohol, vision loss, smoking, and hearing loss (PAF = 44.6%), and excessive alcohol, diabetes, vision loss, and hearing loss (PAF = 43.1%).

**Table 6:** The relationship between risk factor clusters and dementia risk: Cox proportional-hazards model.

Cluster	Categories included in the cluster	Participants		HR (95% CI)	*p*
		Dementia	Participants		
	No coexistence of risk factors.	222	4,184	1.00 (Reference)	
1	**Excessive alcohol (96.5%)** **Diabetes (94.0%)** **Vision loss (93.6%)** **Hearing loss (90.3%)**	**447**	**1837**	**4.11 (3.42, 4.95)**	**<0.001**
2	Obesity (100.0%) Smoking (96.9%) Excessive alcohol (87.6%) Depression (87.3%)	210	1728	2.67 (2.21, 3.23)	<0.001
3	Low education (100.0%) Diabetes (100.0%) Depression (98.2%) Obesity (97.8%)	91	1872	0.97 (0.76, 1.24)	0.81
4	Vision loss (100.0%) Diabetes (99.9%) Hearing loss (97.6%) Smoking (95.2%)	316	3,798	1.40 (1.18, 1.66)	<0.001
5	**Excessive alcohol (96.3%)** **Vision loss (90.2%)** **Smoking (84.3%)** **Hearing loss (83.9%)**	**302**	**1,566**	**5.18 (4.30, 6.23)**	**<0.001**
6	Smoking (94.2%) Excessive alcohol (93.9%) Hearing loss (91.0%) Low education (88.7%)	237	3,311	1.42 (1.18, 1.71)	<0.001
7	Smoking (100.0%) Hearing loss (97.0%) Excessive alcohol (95.6%) Diabetes (92.0%)	413	2,185	3.76 (3.20, 4.43)	<0.001
8	**Excessive alcohol (98.6%)** **Obesity (96.5%)** **Diabetes (82.5%)** **Smoking (81.4%)**	**614**	**2,152**	**5.96 (5.11, 6.95)**	**<0.001**
9	Obesity (99.4%) Excessive alcohol (97.9%) Diabetes (97.0%) Hearing loss (96.4%)	558	3,949	2.57 (2.20, 3.01)	<0.001

Adjusted for age, sex, stroke and heart disease. Bold values indicate the three clusters with the highest dementia risk ratios.

## 4 Discussion

This study indicates that low educational attainment is the most significant factor contributing to an increased risk of dementia and accelerated cognitive decline, followed closely by depression and hearing loss. The risk of dementia shows a clear dose–response relationship with the number of modifiable risk factors, and the association between dementia risk and different clusters of risk factors varies considerably. This point is also validated by the PAF calculated for both individual risk factors and clustered risk factors.

Prior research has established a significant correlation between education level and dementia risk, with one study illustrating that higher education levels are associated with a 27% increase in survival time for individuals without dementia when compared to those with lower educational attainment (16). This may be associated with higher education levels facilitating greater neuronal connectivity and synaptic stimulation in the brain, thereby enhancing neural plasticity and increasing the network density of the cerebral cortex. Prolonged cognitive activities, such as learning and education, can increase the number of synaptic connections. The enhancement of synaptic density is closely linked to elevated levels of brain-derived neurotrophic factor (BDNF), a critical molecule that supports neuronal survival, synaptogenesis, and plasticity (39, 40). A study conducted in China revealed that the incidence and prevalence of dementia markedly increased among populations with lower education levels (41). Furthermore, a study involving Asian Americans reported that individuals with higher education levels are linked to a reduced risk of dementia (42). In our study, a greater proportion of participants in the CHARLS cohort exhibited lower education levels and a correspondingly higher incidence of dementia. This relationship is linked to the rising life expectancy of older adults in China, coupled with the lower educational attainment experienced by participants earlier in their life course (43). In developing nations, education represents one of the most critical modifiable risk factors, making it imperative to address educational attainment (44, 45).

Hearing loss has been identified as being associated with dementia and cognitive decline across all participant cohorts. Prior meta-analyses have documented a significant association between hearing loss and subsequent dementia (46–48). The Health ABC study revealed that older adults experiencing hearing loss faced a 55% increased risk of developing dementia (49). The Baltimore Longitudinal Study of Aging identified a significant correlation between the severity of baseline hearing loss and the risk of dementia (50). Hearing loss can lead to loneliness, depression, and social isolation, which interact with brain pathology. Hearing impairment may result in irreversible damage, subsequently disrupting cortical activity in the brain and potentially triggering the onset of dementia (51).

Depression is associated with dementia and cognitive decline in middle-aged and older adults, aligning with previous research that indicates a relationship between depression and both dementia and cognitive function. A cohort study in Denmark found that depression in middle or late life is significantly associated with increased dementia risk Elser (52). A cohort study in the UK reported a 51% increase in dementia risk among individuals aged 50–70 with baseline depression (53). Firstly, both depression and dementia are classified as mental health disorders and may share common pathological features. Secondly, depression can increase cortisol secretion, leading to hippocampal atrophy or triggering inflammatory responses (54). Therefore, treatment for depression may help slow down or prevent the onset of dementia (55).

In summary, education level, depression, and hearing loss are significant risk factors influencing dementia and cognitive ability. Therefore, it is essential to enhance educational opportunities early in life, promote cognitive stimulation throughout the life course to maintain a high level of cognitive reserve, and focus on mental health and living conditions to delay or prevent the onset of dementia.

Hypertension is likely to cause cortical white matter lesions and microvascular damage in the brain, which can subsequently lead to cognitive decline and the onset of dementia (56). Hypertension appeared to elevate the risk of dementia among participants; however, the statistical significance was weak in the overall cohort as well as in the HRS and ELSA studies. In high-income countries such as the United States and the United Kingdom, elevated levels of socioeconomic development may contribute to increased blood pressure in middle and late life, which could partially explain the weak associations observed in these cohorts (5).

Smoking is closely associated with the biological mechanisms of dementia, primarily through inducing oxidative stress and chronic inflammatory responses, which lead to neuronal damage and pathological changes in the brain, thereby increasing the risk of dementia. Additionally, smoking damages cerebrovascular health, resulting in insufficient blood supply to the brain and ischemic injuries. Over time, this can cause brain atrophy, further exacerbating cognitive decline. These changes are strongly linked to dementia types such as Alzheimer’s disease (57). Prior studies have demonstrated a significant correlation between midlife or current smoking and an elevated risk of dementia (58–60). In our study, smoking was linked to an increased risk of dementia and cognitive decline among participants in the overall cohort, HRS, CHARLS, and ELSA; however, the statistical significance was less pronounced in the overall cohort. This discrepancy may stem from the diversity of socioeconomic backgrounds, ethnicities, and lifestyles among participants, which could mask the true impact of smoking on dementia risk and result in attenuated statistical correlations. Furthermore, the method of assessing smoking—such as self-reporting—may introduce bias, potentially leading to an underestimation of the true impact of smoking behavior on dementia risk.

In this study, obesity was found to correlate with an elevated risk of dementia, supporting previous research that connects midlife and late-life obesity with a heightened risk of dementia (12). Obesity is associated with increased cortisol levels, inflammation, and negative health outcomes, which may contribute to the development of dementia (54). Nonetheless, some studies suggest that obesity in late life may be linked to a decreased risk of dementia (61). Our findings revealed that obesity was connected to a deceleration in cognitive decline across the overall cohort, including HRS and ELSA; in CHARLS, obesity similarly correlated with a slowdown in cognitive decline. This phenomenon may stem from the association between obesity and cognitive reserve, along with elevated levels of neurotrophic factors (62). Furthermore, the prevalence of obesity among older adults in China remains relatively low, at just 4.7%. In conclusion, while obesity is a modifiable risk factor for dementia, its impacts on dementia risk and cognitive abilities necessitate further exploration within specific national and social contexts.

Prior high-quality studies involving long-term follow-up research have demonstrated that physical activity can mitigate the risk of developing dementia. High levels of physical activity contribute to cognitive function, potentially by altering cerebral blood flow and increasing brain-derived neurotrophic factor (BDNF), ultimately enhancing brain plasticity and reducing neuroinflammation (63). A meta-analysis indicated that greater levels of physical activity correlate with lower incidence rates of dementia, implying that the beneficial impact of physical activity on dementia may necessitate both increased intensity and extended duration to manifest significant effects (64, 65). In this study, we observed that a deficiency in physical activity may elevate the risk of dementia; however, the statistical significance was weak within the overall cohort and HRS, while it was more pronounced in ELSA. Conversely, in CHARLS, a deficiency in physical activity correlated with a reduced risk of dementia, although this association was similarly weak. This discrepancy may stem from the diverse socioeconomic and cultural backgrounds of participants across the three cohorts, which could affect physical activity levels and health outcomes (66). Furthermore, the relatively brief follow-up duration in this study may also account for the observed weak statistical significance.

Excessive alcohol consumption induces oxidative stress and neuroinflammation, leading to neuronal damage and thereby increasing the risk of dementia. Furthermore, chronic heavy drinking can cause brain atrophy and disturbances in the neurotransmitter system, further exacerbating cognitive decline and increasing the likelihood of developing dementia (67). Previous cohort studies have indicated that heavy drinking correlates with an 8% increase in the risk of dementia (68). Conversely, our study revealed a negative correlation between excessive alcohol consumption and both dementia risk and cognitive decline. This finding contrasts with previous research. One potential explanation for this discrepancy is our definition of excessive drinking, which encompasses consumption more than half the week, including drinking 4–6 days per week, daily, or more than twice a day; all these data were self-reported and may introduce selection bias. Moreover, it is conceivable that participants consumed alcohol prior to the survey but ceased by the time of data collection, or they may not have been drinking during the survey period but resumed during follow-up. This could elucidate the observed negative correlation between excessive drinking and dementia risk and cognitive decline, necessitating further investigation.

This study additionally revealed that diabetes and social isolation are linked to an elevated overall risk of dementia and accelerated cognitive decline. Prior research has demonstrated a significant correlation between the age of onset of type 2 diabetes and the risk of developing dementia (69). Diabetes can lead to metabolic changes in the brain, resulting in increased β-amyloid toxicity, tau protein hyperphosphorylation, and neuroinflammation, all of which contribute to an increased risk of dementia (69). Furthermore, the World Health Organization recognizes diabetes as one of the detrimental factors affecting dementia across the life course (70). Previous meta-analyses indicate that regular social contact can mitigate the risk of dementia in older adults, while those with higher levels of social isolation encounter a greater risk (71, 72). Regular and extensive social interactions can foster cognitive reserve, alleviate loneliness, and encourage healthy behaviors, thereby decelerating cognitive decline and decreasing the incidence of dementia (73).

Vision loss may lead to reduced sensory input, increased cognitive load, and consequently affect brain structure and function (74). In this study, vision loss was significantly linked to accelerated cognitive decline. Prior research has similarly indicated significant correlations between vision loss, dementia, and cognitive abilities (75, 76). In our study, vision loss was found to elevate the risk of dementia among participants; however, the statistical significance was weak. This may be attributed to survival bias or limitations in the definitions and baseline assessments of modifiable risk factors, which might not adequately capture changes over the life course.

In conclusion, our findings suggest that, with the exception of excessive drinking, the impacts of the other ten risk factors on dementia and cognitive decline among all participants remain consistent.

Beyond evaluating individual modifiable risk factors, this study also explored the relationship between the quantity of modifiable risk factors and dementia risk among all participants. Older adults in China displayed the highest prevalence of coexisting risk factors, potentially linked to the limited medical and caregiving resources available to the aging population, as well as the suboptimal living conditions encountered earlier in their life course (77). Our findings demonstrate that the risk of dementia significantly escalates with the number of risk factor events, a result that aligns with observations from a comparable meta-analysis (78). Various risk factors may interact through biological pathways, intensifying neurodegenerative changes. For instance, biological processes like oxidative stress and neuronal damage may be exacerbated by the presence of coexisting risk factors, ultimately contributing to an elevated risk of dementia (79, 80).

A dose–response relationship was observed between modifiable dementia risk factors and dementia risk. Various clusters of risk factors have different associations with dementia risk; the clusters of excessive alcohol, diabetes, vision loss, and hearing loss; excessive alcohol, vision loss, smoking, and hearing loss; and excessive alcohol, obesity, diabetes, and smoking were associated with three times or more the risk of dementia. The clustering of these risk factors may reflect shared pathophysiological mechanisms, including the synergistic effects of vascular damage, chronic inflammation, and metabolic disorders. Specifically, the clusters of excessive alcohol, diabetes, vision loss, and hearing loss, as well as excessive alcohol, vision loss, smoking, and hearing loss, may collectively induce neuroinflammation and oxidative stress responses. These processes can accelerate neurodegenerative changes and increase the risk of dementia. Additionally, these clusters contribute to elevated stress and cognitive load, rendering individuals more vulnerable to dementia (57, 67, 81, 82). For the cluster comprising excessive alcohol, obesity, diabetes, and smoking, these risk factors are likely to jointly trigger systemic inflammation and oxidative stress, further impairing brain health. These processes not only accelerate neurodegenerative changes, increasing dementia risk, but also cause vascular damage, thereby disrupting cerebral blood flow and elevating the risk of vascular dementia (54, 57, 67, 69). Notably, excessive alcohol consumption appears consistently in all high-risk combinations, suggesting that alcohol may play a pivotal role in the interaction among multiple risk factors.

Our findings are consistent with a large cohort study in the UK, which found that multiple diseases are associated with an increased risk of dementia and that the coexistence of diseases poses a greater hazard for dementia (31). Our findings on the coexistence of multiple risk factors suggest that greater attention should be paid to the presence of multiple risk factors rather than focusing solely on individual ones. The different combinations of risk factors indicate the need for developing tailored prevention strategies targeting specific risk factor combinations. In this study, the cluster of low education, diabetes, depression, and obesity was not associated with dementia risk, possibly due to insufficient statistical power resulting from a limited sample size of dementia cases. However, when these factors coexist with other risk factors, they exhibit significant synergistic effects, reflecting a complex network of interactions among the risk factors.

The strengths of this study lie in its large sample size and the incorporation of three extensive, nationally representative cohorts, which facilitate long-term follow-up and comprehensive comparative analysis. Additionally, we integrated modifiable risk factors into the analysis of event frequency and coexistence associated with dementia risk. Nevertheless, our study has certain limitations. Although we adjusted for confounding variables, residual confounders—such as the APOE ε4 genotype and socioeconomic status—may persist. This study did not analyze the impact of changes in risk factors during follow-up on dementia and cognitive decline. Moreover, the majority of the modifiable dementia risk factors and covariates utilized in this study were self-reported, potentially introducing bias. Future research should strive to diversify and ensure more accurate measurement of risk factors to corroborate our findings.

## 5 Conclusion

In conclusion, modifiable risk factors for dementia are closely associated with an increased risk of dementia onset and accelerated cognitive decline. Multiple risk factors frequently coexist and interact, and dementia risk significantly escalates as the number of modifiable risk factors increases over a lifetime. Our findings emphasize the strengthening effect of coexisting modifiable risk factors on dementia onset, suggesting that more attention should be given to the coexistence of multiple risk factors. As the global number of dementia cases continues to rise, there is an urgent need for more effective strategies to address modifiable risk factors for dementia to prevent and mitigate its onset. Future research should focus on conducting prospective cohort studies targeting young and middle-aged populations to evaluate the combined effects of multifactorial interventions. These interventions may include promoting physical exercise, controlling alcohol consumption, smoking cessation, adopting a healthy lifestyle, and improving education levels. Furthermore, future studies should explore the relationships between risk factors and the mechanisms underlying dementia development. In clinical practice, it is recommended to incorporate multidimensional risk assessment into routine health check-ups and develop integrated intervention models through interdisciplinary collaboration. Early control or simultaneous intervention of multiple risk factors may prove to be a promising approach to reducing the incidence of dementia.

## Data Availability

The datasets presented in this study can be found in online repositories. The names of the repository/repositories and accession number(s) can be found in the article/supplementary material.
